# Novel sustainable synthesis of vinyl ether ester building blocks, directly from carboxylic acids and the corresponding hydroxyl vinyl ether, and their photopolymerization†

**DOI:** 10.1039/c8ra04636k

**Published:** 2018-07-10

**Authors:** Maja Finnveden, Sara Brännström, Mats Johansson, Eva Malmström, Mats Martinelle

**Affiliations:** Department of Industrial Biotechnology, KTH Royal Institute of Technology, AlbaNova University Centre SE-106 91 Stockholm Sweden matsm@kth.se; Department of Fibre and Polymer Technology, KTH Royal Institute of Technology Teknikringen 56-58 SE-100 44 Stockholm Sweden mavem@kth.se

## Abstract

Increased environmental awareness has led to a demand for sustainable, bio-based materials. Consequently, the development of new benign synthesis pathways utilizing a minimum of reaction steps and available bio-based building blocks is needed. In the present study, vinyl ether alcohols and functional carboxylic acids were used to synthesize bifunctional vinyl ether esters using the immobilized enzyme *Candida antarctica* lipase B as a catalyst. Vinyl ethers are attractive alternatives to (meth)acrylates due to low allergenic hazards, low toxicity, and fast polymerization; however, difficult synthesis limits the monomer availability. The synthesis was performed in one-pot and the described method was successful within a broad temperature range (22–90 °C) and in various organic solvents as well as in the bulk. The synthesis of different vinyl ether esters reached high conversions (above 90%) after less than 1 h and products were purified by removing the enzyme by filtration using only small amounts of acetone. This approach is a straightforward route to reach monomers with multiple types of functionalities that can be used as different photo-curable thermoset resins. In this work, this was demonstrated by polymerizing the monomers with cationic and radical UV-polymerization. By changing the functional carboxylic acids, the architecture of the final polymer can be tailored, herein demonstrated by two examples. In the developed versatile method, carboxylic acids can be used directly as acyl donors, constituting a more sustainable alternative to the carboxylic acid derivatives used today.

## Abstract 2

Synthesis of bifunctional vinyl ether ester monomers directly from the corresponding alcohol and carboxylic acid.

## Graphical abstract

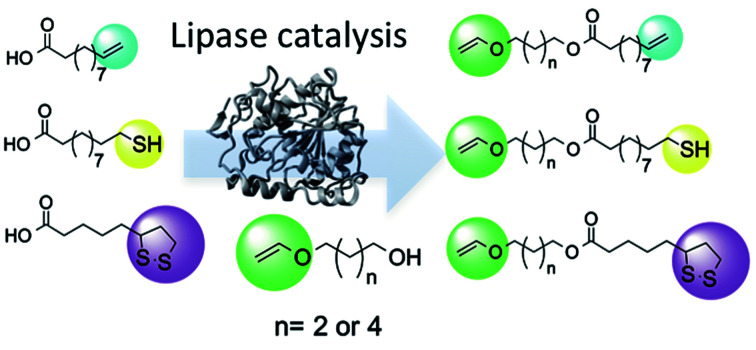

## Introduction

The demand for sustainable polymeric materials is continuously growing. In order to meet these demands, using bio-based resources together with efficient synthetic strategies is important. In particular, processes that minimize the amount of toxic compounds and have low energy consumption are of interest. In this context, photopolymerization is a promising approach; polymerization can be performed under benign reaction conditions with no need for addition of volatile organic solvents, low energy consumption, low temperatures, and often higher reaction rates when compared to thermally initiated processes, which overall results in lower material cost. The properties of the photocured polymers can be tailored to different applications.^1,2^ Thus, monomers that undergo rapid photopolymerization are of great commercial interest and can be found in applications such as photocurable coatings, composites, and printing inks.^2–5^ Today, two main types of photopolymer chemistries are used commercially: free-radical and cationic photo-polymerization. Free-radical photopolymerization is the most used technique for industrial applications. This can be attributed to the wide availability of photoinitiators and the high reactivity and availability of monomers.^6,7^ Especially methacrylate and acrylate monomers have rendered significant interest and commercial importance. Due to the wide range of methacrylate and acrylate monomers and difunctional oligomers available on the market, the structural properties of the resulting polymers can be tailored giving access to materials with a variety of properties.^1^

Despite the wide use, acrylate and methacrylate systems often suffer from allergenic hazards, toxicity and have an unpleasant odor associated with acrylate monomers. Some of the drawbacks can be addressed by using less volatile oligomeric acrylates, but these larger structures are often more difficult to process due to higher viscosity. In addition, most free-radical polymerization systems are sensitive to oxygen inhibition and thus require an inert atmosphere for high polymerization rates and full monomer conversion to be reach.^1,6–8^ Cationic photopolymerization has been shown to be advantageous compared to free-radical polymerization in several aspects.^7^ Oxygen inhibition does not occur for cationic polymerization and furthermore, once initiated, cationically polymerizable monomers, such as epoxides and vinyl ethers, undergo dark polymerization in which they can continue to polymerize without radiation. The most common monomers for cationic photopolymerization are epoxides. Vinyl ethers on the other hand react faster^9^ and are considered attractive alternatives to methacrylates and acrylates, due to their low allergenic hazards, low toxicity, and fast polymerization. The availability of vinyl ether monomers is however substantially lower, limiting the material properties that can be obtained.^2,3^ To overcome the issues associated with free-radical polymerization and utilize the advantages with cationic polymerization, there is a demand for efficient and benign methods that allow easy access to a wider range of vinyl ether functional monomers.

Vinyl ether-terminated esters is a versatile class of functional monomers that can be designed to combine attractive properties such as chemical and mechanical resistance, fast polymerization and adjustable strength. They can be either monomers or oligomers, enabling design of crosslinking-segments to suit various applications.^10^ Starting with hydroxyl vinyl ethers, vinyl ether-terminated esters can be prepared by reaction with carboxylic acid derivatives.

**Scheme 1:**
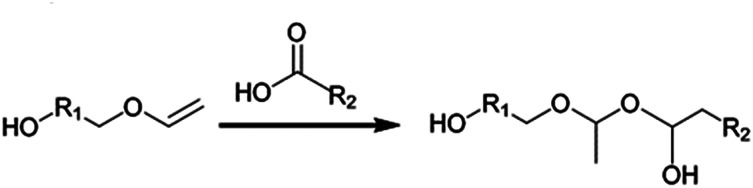
Possible side reaction that takes place if direct esterification is attempted without enzymatic catalysis.

Vinyl ethers have been shown to be very acid-labile and the direct use of a carboxylic acid is therefore not possible when using conventional organic synthesis and catalysts. In the presence of a carboxylic acid, the vinyl ether moiety is susceptible to addition reactions with compounds containing active hydrogen atoms (see Scheme 1).^10,11^ Therefore, vinyl ether esters are synthesized using carboxylic acid derivatives. Commonly used derivatives include acid chlorides or short chain esters (*e.g.* methyl or ethyl). However, using these starting compounds is often associated with disadvantages such as multiple reaction steps or purification issues. For example, when acid chlorides are used, an acid scavenger (such as an amine) must be used to neutralize the formed HCl, since it may otherwise cause hydrolysis or premature polymerization of the acid-labile vinyl ether component.^12^ If an amine scavenger is used, all traces of amine must be removed from the product or it will inhibit the subsequent cationic radiation curing reaction.

Using short chain esters (*e.g.* methyl or ethyl esters) is thus the preferred synthesis strategy. However, using short chain esters involves one additional reaction step to transform the starting carboxylic acids to the corresponding ester, generally requiring removal of water and/or excess amounts of the reactants to get satisfactory conversion. Additionally, a large volume of volatile organic solvent is often used.^13^ Thus, developing a synthetic route that allows for direct esterification of carboxylic acids, without the need for derivatives or scavengers, would be preferred for the synthesis of vinyl ether functional esters. This would for example allow for fatty acids or other carboxylic acids to be used which can make the vinyl ether monomers more bio-based.^14,15^

In this work we present a novel method that can be used to synthesize vinyl ether functional esters directly from carboxylic acids and hydroxyl-functional vinyl ethers without the need for any solvents. This is accomplished using immobilized lipase B from *Candida antarctica* (CalB), an efficient catalyst that largely favors the formation of ester over the inactivation of the acid-labile vinyl ether. Since the favored kinetics of the esterification rapidly lowers the concentration of acidic carboxyl, unwanted side reactions can be avoided. Due to its efficiency and selectivity, CalB has been shown useful in reactions where protection chemistry would normally be utilized to keep functional groups intact.^16–22^ Immobilized CalB works at low temperatures, but it is also able to catalyze reactions at temperatures up to 100 °C in bulk and in organic media.^23–25^ Additionally, immobilization facilitates removal and reuse of the catalyst after reaction.^26^

The synthesis route presented here is versatile and straightforward, providing easy access to a series of bifunctional vinyl ether ester monomers with various additional functional groups. The study was designed to evaluate the applicability of the synthesis method with respect to temperature and solvents, demonstrating a flexible system that can be adapted to the vast amount of carboxylic acids and hydroxyl vinyl ethers currently available.

Combining the method presented here with recently promising results towards the synthesis of vinyl ethers through environmentally viable routes^27–29^ and new photoinitiators for polymerizing vinyl ethers, as reviewed by Sangermano *et al.*,^2^ adds for a new benign way to reach a wide range of vinyl ether functional building blocks.

## Experimental

### Materials

*Candida antarctica* lipase B (CalB) immobilised on an acrylic carrier, >5000 U g^−1^ (Novozyme 435), 10-undecenoic acid (UA), 11-mercaptoundecanoic acid (MUA), lipoic acid (LA), and 1,4-butanediol vinyl ether (BVE) were supplied by Sigma Aldrich. 1,6-Hexanediol vinyl ether (HVE) was supplied by BASF. All chemicals were used as received unless otherwise noted. Novozyme 435 was stored in a desiccator with saturated LiCl solution. The hydroxyl vinyl ether: BVE, and HVE, as well as solvents were stored with 4 Å molecular sieves.

### Characterization

^1^H-NMR and ^13^C-NMR spectra were recorded on a Bruker AM 400 MHz instrument. FTIR spectra were recorded on a Perkin-Elmer Spectrum 2000 FTIR instrument (Norwalk, CT) equipped with a single reflection (ATR: attenuated total reflection) accessory unit (Golden Gate) from Graseby Specac LTD. Size exclusion chromatography (SEC) was performed on a Malvern VISCOTEK GPCmax equipped with a refractive index detector. Differential scanning calorimetry (DSC) was performed with a Mettler Toledo differential scanning calorimeter DSC 820. For more details please see the ESI.†

### Lipase catalyzed acylation of hydroxyl vinyl ether

#### Study of reaction conditions

The synthesis of 6-(vinyloxy)hexyl 10-undecenoate (HVEUA) was performed in different solvents (bulk, toluene, acetonitrile (ACN), methyl *tert* butyl ether (MTBE) and 2-methyltetrahydrofuran (Me-THF)) and at different temperatures (22, 60 and 90 °C). 10-Undecenoic acid (UA) and 1,6-hexanediol vinyl ether (HVE) were added in a 1 : 1 molar ratio, see Table S1† for amounts of UA and HVE. The reactions were started by the addition of 10 wt% immobilized CalB. Consecutive samples were withdrawn, filtered through a cotton filter and analyzed with GC. Endpoint samples were further analyzed by ^1^H-NMR. All the reactions performed with HVE were also performed with 1,4-butanediol vinyl ether (BE). More details can be found in the ESI.†

#### Synthesis of 6-(vinyloxy)hexyl 10-undecenoate (HVEUA)

HVE (0.39 g, 2.7 mmol) and UA (0.50 g, 2.7 mmol) were placed in a round bottom flask equipped with a magnetic stirrer and the reaction was started by adding immobilized CalB (90 mg). Reactions were run at ambient temperature or the flask was placed in a preheated oil bath set to 60 or 90 °C. For the reactions performed at ambient temperature, 4 Å molecular sieves were added. The reaction was stopped after 1 h at which point the product was dissolved in acetone and the immobilized enzyme was filtered off. The product was recovered by evaporating the acetone.

#### Synthesis of 4-(vinyloxy)butyl 10-undecenoate (BVEUA)

Synthesis of BVEUA used the same conditions as for HVEUA but with 3.4 mmol of UA and BVE.

#### Synthesis of 6-(vinyloxy)hexyl 11-mercaptoundecanoate (HVEMUA)

HVE (0.39 g, 2.7 mmol), MUA (0.59 g, 2.7 mmol) and 5 mL toluene were placed in a round bottom flask equipped with a magnetic stirrer and 4 Å molecular sieves. The reaction was started by adding immobilized CalB (100 mg) and was performed at ambient temperature. Product was recovered in the same way as HVEUA.

#### Synthesis of 4-(vinyloxy)butyl 11-mercaptoundecanoate (BVEMUA)

Synthesis of BVEMUA used the same conditions as for HVEMUA but with 3.4 mmol of MUA and BVE.

#### Synthesis of 6-(vinyloxy)hexyl lipoate (HVELA)

HVE (0.41 g, 2.9 mmol) and LA (0.59 g, 2.9 mmol) were placed in a round bottom flask equipped with a magnetic stirrer. Immobilized CalB (100 mg) was added and the flask was placed in a pre-heated oil bath set to 60 °C. After about 20 minutes, the pressure was reduced and the reaction was run in vacuum. Product was recovered in the same way as HVEUA.

#### Synthesis of 4-(vinyloxy)butyl lipoate (BVELA)

Synthesis of BVELA used the same conditions as for HVELA but with 3.4 mmol of LA and BVE.

### Polymerization and polymerization kinetics

UVAcure 1600 was used as a photoinitiator for cationic polymerization and Irgacure 651 was used as radical photo initiator. Monomers (100 mg), was mixed with photoinitiator (1 mg) in vials and covered with aluminium foil. Polymerization was performed in vials with a magnetic stirrer, using a Hamamatsu L5662 UV-lamp (40 mW cm^−2^) and irradiated until VEs were consumed or the reaction stagnated. Kinetics of the radical and cationic polymerizations of the synthesized monomers was studied with real time-FTIR using an UV intensity of 17 mW cm^−2^. The polymeric products were analyzed by: ^1^H-NMR, FTIR, THF-SEC and DSC.

## Results and discussion

### Synthesis of vinyl ether esters through enzyme catalysis

**Scheme 2:**
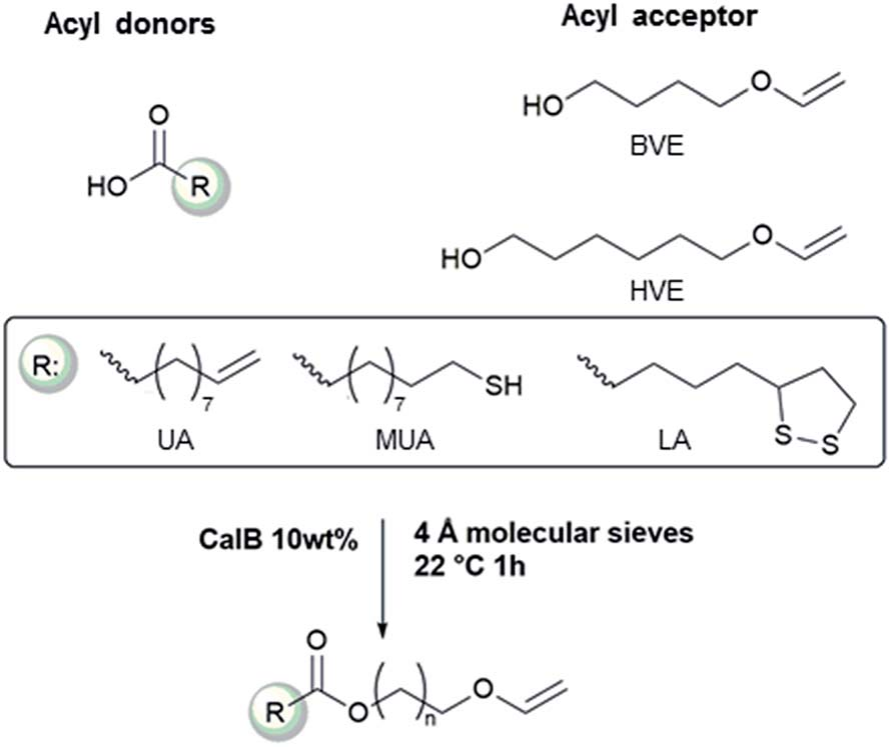
The fatty acids and alcohol vinyl ethers used in this study to make vinyl ether functional ester monomers.

The acid-lability of vinyl ethers renders it impossible to synthesize vinyl ether functional esters directly from carboxylic acids, using conventional catalysis, without detrimental side reactions (Scheme 1). The aim of the present study was therefore to develop a synthetic pathway utilizing enzyme catalysis where the esterification is kinetically very favored over any side reaction. Scheme 2 shows the acyl donors and acyl acceptors used in this investigation. The study was designed to evaluate the applicability of the synthesis method with respect to temperature and solvents, demonstrating a flexible system that can be adapted to a wide range of carboxylic acids and hydroxyl vinyl ethers currently available.

**Fig. 1:**
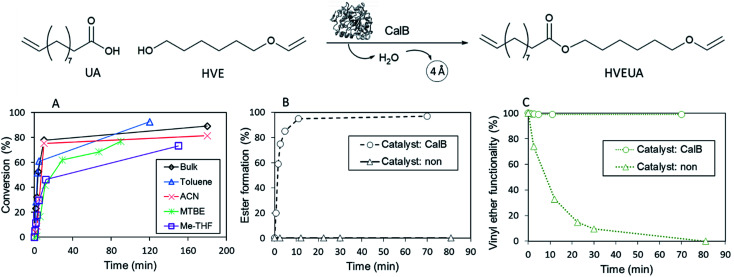
Reaction profiles for the synthesis of 6-(vinyloxy)hexyl 10-undecenoate (HVEUA) by lipase catalyzed acylation of 6-hydroxyhexyl vinyl ether (HVE) with 10-undecenoic acid (UA). (A) High conversions were reached quickly in several different solvents at 22 °C, as evaluated with GC. (B) Ester formation at 90 °C through ^1^H-NMR. Catalyzed (○) and uncatalyzed (Δ). (C) Intact vinyl ether functionality at 90 °C through ^1^H-NMR. Catalyzed (○) and uncatalyzed (Δ). Note: in the uncatalyzed reaction HVE was mixed with UA.

The reaction conditions for the lipase-catalyzed synthesis of vinyl ether-terminated esters were studied using 10-undecenoic acid (UA) and 1,6-hexanediol vinyl ether (HVE) (Fig. 1). All reactions were also repeated using 1,4-butanediol vinyl ether (BVE) as an acyl acceptor (Fig. S1 and S2, ESI†).

The influence of reaction media at 22 °C was investigated using bulk, toluene, methyl *tert*-butyl ether (MTBE), 2-methyltetrahydrofuran (Me-THF) and acetonitrile (ACN) (Fig. 1). The conversion was followed by gas chromatography (GC) and calculated from the consumption of the HVE. Additionally, end-point samples were analyzed by ^1^H-NMR to confirm monomer conversion and that the vinyl ether functionality, in the product HVEUA, remained intact. The reaction profiles, (Fig. 1A, S1 and Table S4†), revealed that the synthesis was possible in solvents with different properties. However, the synthesis in bulk and toluene reached the highest conversions. This is in agreement with previous studies on lipase catalysis in different organic solvents.^30,31^ In general, enzyme activities in polar organic solvents are lower than in nonpolar solvents because the polar ones, to a high degree, strip essential water from the enzyme.

Furthermore, the reaction kinetics were studied at 90 °C. The reaction profiles for both the uncatalyzed and catalyzed reactions are shown in Fig. 1B (denoted (○) and (Δ) respectively) and 1C. In Fig. 1B the reaction profile for the ester formation is shown. The uncatalyzed reaction was followed with ^1^H-NMR for circa 80 min and during this time, no ester formation was observed without the addition of the catalyst. In Fig. 1C the percentage of intact vinyl ether functionality during the same time interval is shown and confirms the acid-lability of the vinyl ether. ^1^H-NMR spectra of reactions at 90 °C, after circa 80 min revealed that the vinyl ether functionality was intact for the CalB-catalyzed reaction, whereas it was lost completely during the uncatalyzed reaction. When CalB was added as a catalyst, the reaction was run to completion after 10 min. This shows that the addition of a catalyst that kinetically favors ester formation, rapidly decreasing the acid concentration, is crucial to keep the vinyl ether moiety intact. The results were confirmed by reproducing the experiments with BVE (Fig. S1 and S2 in ESI†).

Catalyst-free reactions and reactions with other conventional catalysts were also explored. No ester formation was observed for any of the catalyst-free reactions (in Fig. 1B and C, the reaction profile for uncatalyzed reaction at 90 °C is shown (Δ)). In addition, catalyst-free reactions at 60 and 22 °C were studied. At 60 °C 40% of the vinyl ether functionality was gone after 1 h, and after 24 h, the vinyl ether was completely consumed. Uncatalyzed reaction at 22 °C left overnight showed over 60% loss of the vinyl ether functionality (analyzed by ^1^H-NMR).

Additionally, the condensation of BVE and UA was attempted with the organometallic catalyst titanium(iv)butoxide (Ti(OBu)_4_) at 160 °C and the organobase, 1,5,7-triazabicyclo[4.4.0]dec-5-ene (TBD), but no formation of the bifunctional vinyl ether-terminated esters was observed. For the reaction at 160 °C catalyzed by Ti(OBu)_4_, all vinyl ether functionality was gone after 2.5 min (Fig. S5, ESI†).

To expand the method, vinyl ether-terminated esters were synthesized in gram scale, using two additional carboxylic acids: 11-mercaptoundecanoic acid (MUA) and lipoic acid (LA) (Table 1). MUA was chosen as carboxylic acid to present a direct route to thiol-vinyl ether functional building blocks viable for thiol–ene systems. Additionally, LA was chosen to provide a stable thiol that can be synthesized without the addition of a radical scavenger. Neither MUA nor LA were soluble in the hydroxyl vinyl ethers at 22 °C, therefore toluene was used as a solvent for MUA. It should also be noted that it was necessary to add a radical inhibitor to the reaction with MUA to prohibit spontaneous thiol–ene reaction, even when the reaction was performed at ambient temperature. This was not the case for LA which could be used in bulk, but required temperatures above 60 °C which is LA's melting point. The results shown in Table 1, were obtained after 1 h reaction time and show high conversions for all the carboxylic acids used. This straightforward route to produce telechelic vinyl ether monomers from hydroxyl vinyl ethers and carboxylic acids can be expanded to utilize numerous of the readily accessible and versatile renewable fatty acids. It should be mentioned that the choice of carboxylic acids is limited by their p*K*_a_. Previously reported results show a transition at a p*K*_a_ of 4.8, below which the enzyme becomes inactive.^32^

**Table 1:** Vinyl ether terminated esters prepared from the corresponding alcohol and a series of carboxylic acids. The conversion was calculated with ^1^H-NMR (spectra can be found in Fig. 3, S3, S8 and S9 in the ESI)

Vinyl ether functional ester	Conversion (%)
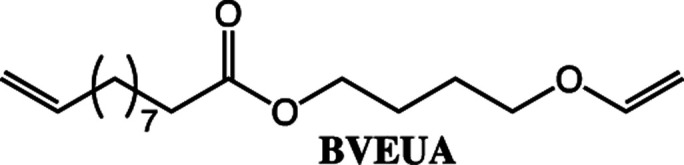	92
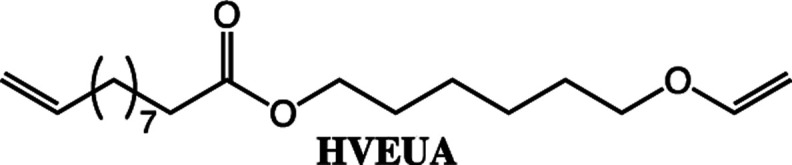	96
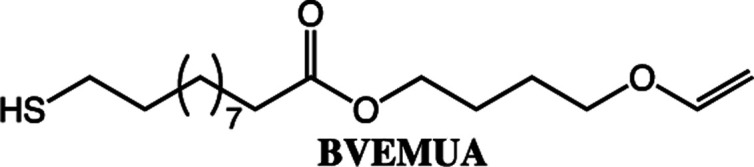	99
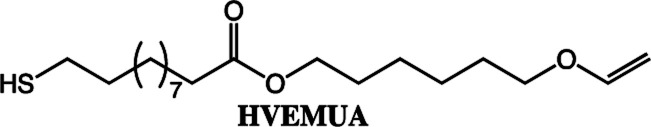	99
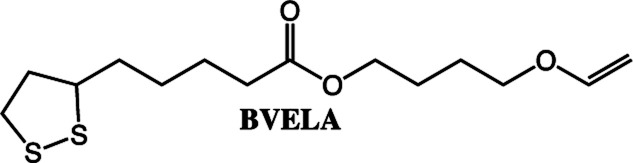	91
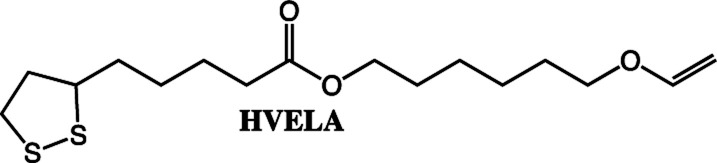	92

### Radical and cationic photopolymerization of vinyl ether ester building blocks

**Fig. 2:**
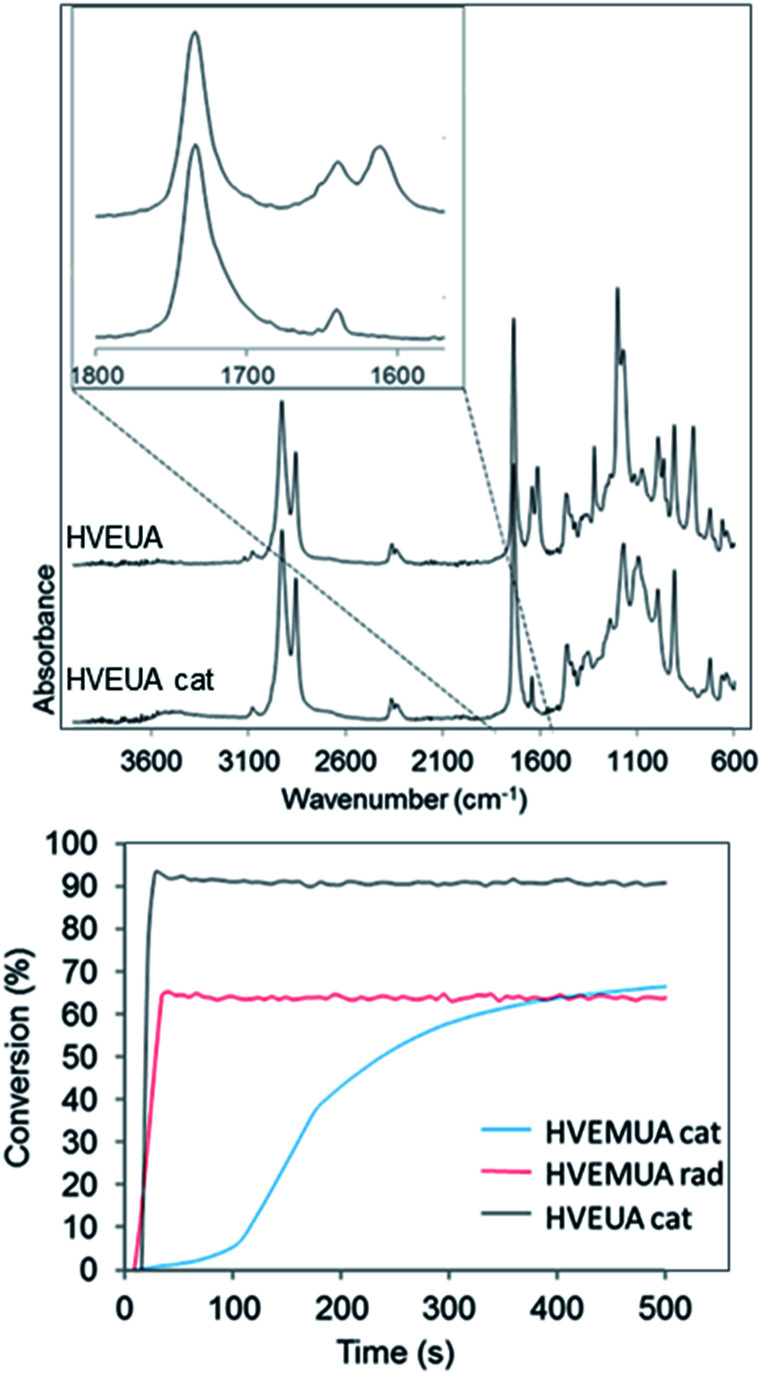
Top: FTIR spectra before and after polymerizing HVEUA with a cationic initiator. Bottom: conversions of the polymerizations studied with RT-FTIR. The conversion was calculated by following the area of the vinyl ether functionality at 1613 cm^−1^. The other FTIR spectra of before and after reaction can be found in the ESI (Fig. S8 and S9†).

**Fig. 3:**
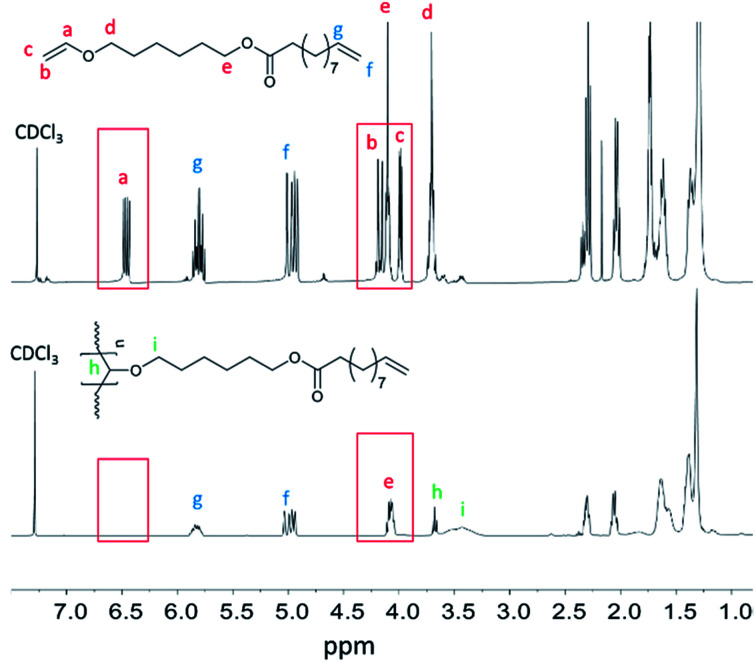
^1^H-NMR of the HVEUA monomer synthesized with enzymatic catalysis (top) and after polymerization of HVEUA with cationic polymerization (bottom).

**Scheme 3:**
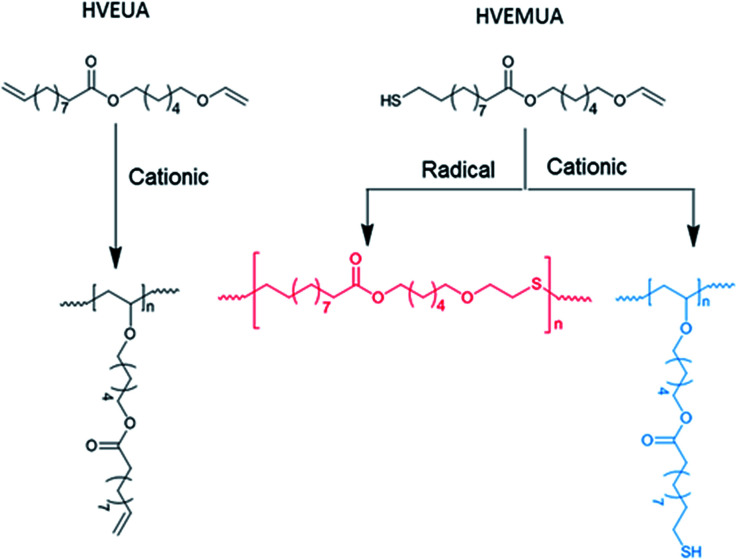
Proposed structures of the formed polymers: radical polymerization of HVEUA was not successful, cationic polymerization of HVEUA renders a linear polymer with pendant –ene groups, radical polymerization of HVEMUA renders a linear polymer and cationic polymerization of HVEMUA renders a linear polymer with pendant thiols.

By polymerizing the bifunctional vinyl ether monomers in Table 1 it should be possible to achieve polymers with different architectures depending on the choice of polymerization route. HVEUA and HVEMUA were polymerized with radical and cationic photo initiation and the reactions were followed with RT-FTIR to study the kinetics and the monomer conversions (Fig. 2). As can be seen, HVEUA reaches high conversion quickly when polymerized cationically, and follows similar kinetics compared to previous studies on cationic polymerization of vinyl ethers.^33,34^ On the other hand when a radical initiator was used, the vinyl ether remained completely unreacted. It is known that vinyl ethers do not homopolymerize by free-radical mechanism, but can copolymerize with electron accepting monomers, like maleates or thiols. This is clearly seen when HVEMUA is radically polymerized; the reaction was run to completion in 30 s. This is to be expected since vinyl ethers (together with norbornenes) have been rated as the most reactive ene functional groups for thiol–ene chemistry.^26^ However, even though the radical polymerization of HVEMUA was very fast, the conversion only reached about 65%. This occurred since the reaction was performed at ambient temperature and it was found that the product was vitrified due to crystallization at this temperature. Cationic polymerization of HVEMUA was much slower than that of HVEUA and we postulate that this is due to the presence of the thiol causing some side reactions or interferences. The structure of HVEUA before and after polymerization can be seen in Fig. 3, and the others can be found in the ESI (Fig. S8 and S9†). As seen in Fig. 3, the vinyl ether has reacted completely and the alkene from the fatty acid is still intact. This could be used to further functionalize the polymer with for example thiol–ene click chemistry. The alkene is also visible in the FTIR spectra shown in Fig. 2. The ^1^H-NMR spectrum of the cationically polymerized HVEMUA (Fig. S9†) shows that mainly cationic polymerization occurred. However, products from radical polymerization can also be seen to some extent and a peak at 4.7 ppm corresponding to an acetal or a thioacetal can also be observed. The proposed structures of the formed polymers can be seen in Scheme 3.

**Fig. 4:**
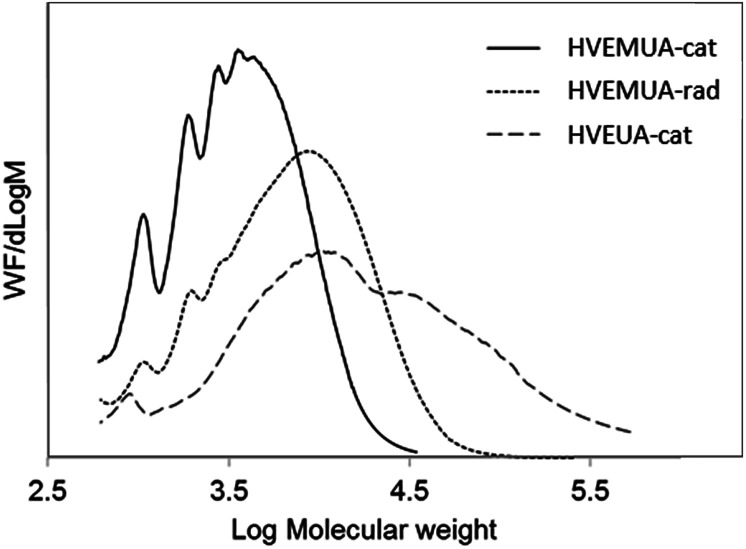
Molecular weight distribution from THF-SEC, showing that HVEMUA cat has the lowest molecular weight, HVEMUA rad has a higher molecular weight and HVEUA cat has the highest and broadest molecular weight.

The molecular weights of the resulted polymers were assessed using THF-SEC and the results can be seen in Fig. 4. The polymer from HVEMUA obtained by cationic polymerization has the lowest molecular weight. This can be explained by the low conversion as seen in the RT-FTIR studies and the observed side reactions (see Fig. S9† for ^1^H-NMR spectra). The molecular weight distribution even appears to have oligomeric resolution indicating that there are lower molecular weight compounds. These could both be oligomers formed with cationic polymerization that have been inhibited due to the presence of thiols or it is the product from various side reactions. Radical polymerization of HVEMUA resulted in a polymer with a somewhat higher molecular weight but the molecular weight is still lower when compared to the polymer made from HVEUA. This can also be related to the RT-FTIR measurements where this polymerization also did not reach full conversion.

Cationic polymerization of HVEUA yielded a polymer with higher molecular weight units and this also relates well to the results from the RT-FTIR studies where it was seen that it reached a much higher conversion than the other two reactions. The molecular weight distribution is also very high but this is not unreasonable since this was not a controlled polymerization.

**Fig. 5:**
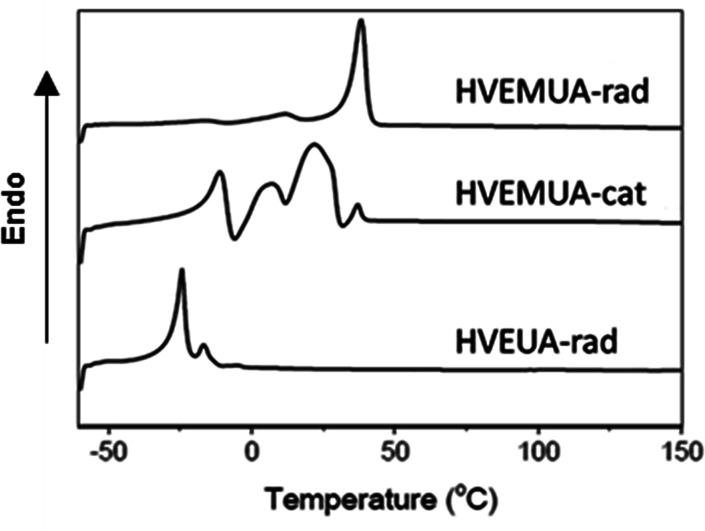
DSC thermograms of the polymers, measured for the second heating cycle of the polymers.

The polymers that were successfully synthesized from the monomers HVEMUA and HVEUA were studied with DSC to determine their thermal properties. The thermograms for the respective monomers can be seen in Fig. 5. As can be seen the polymer from radical polymerization of HVEMUA has a higher crystallization temperature than the others. This is proposed to be the reason why high monomer conversion and higher molecular weights are not reached. From the thermogram it can be seen that the polymer has a crystallization temperature at approximately 40 °C which is above the reaction temperature which is why there is vitrification. The polymer from cationic polymerization of HVEMUA has a distribution of crystallization temperatures.

This is proposed to be due to side reactions yielding different products, which have different melting-temperatures. The cationic polymerization of HVEMUA is slower and does not reach as high conversions as cationic polymerization of HVEUA. The thermal properties of HVEMUA cat in Fig. 5 appears to be have a combination of the melting properties from both HVEMUA-radical and HVEUA-cationic. The lower melting temperature of the polymer of HVEUA formed cationically is reasonable and can be explained by the fact that the repeating units has pendant groups from 10-undecenoic acid.

Further studies will be performed on the polymerization of the lipoate compounds (BVELA and HVELA), however, outside the scope of this paper. In a previous study by Tang *et al.* lipoic acid and 2-hydroxyethyl acrylate were used to synthesize a compound containing two types of radically polymerizable groups (1,2 dithiolane and vinyl).^35^ The compound was polymerized either by using a radical source alone or by first reducing the lipoate compound leading to an AB2-type monomer. The lipoate compounds (HVELA and BVELA) can be expected to behave in the same manner.

## Conclusions

In conclusion, we have developed an efficient method for direct synthesis of vinyl ether-esters monomers based on functional carboxylic acids and hydroxyl-functional vinyl ether monomers. The esterification was conducted under benign reaction conditions from carboxylic acids and alcohol vinyl ethers using immobilized CalB as a catalyst. This direct synthesis of vinyl ether esters is much more sustainable and easy compared to previous methods that both require several reaction steps and difficult purification. Since the enzyme is an efficient esterification catalyst, the ester formation is so fast that the acid content is rapidly decreased before unwanted addition reactions occur with the acid sensitive vinyl ether. The potential to use solvents with different characteristics and a wide temperature range makes it possible to use a large variety of carboxylic acids. For example, multifunctional carboxylic acid derivatives may be used to produce multifunctional vinyl ether products, for the synthesis of starting compounds for rapid photopolymerization. Lipases display chemo, regio- and enantioselectivity, enabling the synthesis of more complex ester monomers for use in material design.

## Conflicts of interest

There are no conflicts to declare.

## Supplementary Material

RA-008-C8RA04636K-s001
