# Supplementary material for: Novel sustainable synthesis of vinyl ether ester building blocks, directly from carboxylic acids and the corresponding hydroxyl vinyl ether, and their photopolymerization
Source: RSC Adv. 2018 Jul 10;8(44):24716–23. doi: 10.1039/c8ra04636k (PMC9082454; doi:10.1039/c8ra04636k)
Supplement: RA-008-C8RA04636K-s001 [file RA-008-C8RA04636K-s001.pdf]

Electronic Supplementary Material (ESI) for RSC Advances.
This journal is © The Royal Society of Chemistry 2018

Electronic Supporting Information

Novel Synthesis of Vinyl Ether Ester Building Blocks, Directly From Carboxylic Acids and the Corresponding Hydroxyl Vinyl Ether and their Photopolymerization

Maja Finnveden[a], Sara Brännström[b], Mats Johansson[b], Eva Malmström[b]* and Mats Martinelle[a]*

[a] Department of Industrial Biotechnology, KTH Royal Institute of Technology, AlbaNova University Centre, SE-106 91 Stockholm, Sweden

[b] Department of Fibre and Polymer Technology, KTH Royal Institute of Technology, Teknikringen 56-58, SE-100 44 Stockholm, Sweden

Correspondence to: matsm@kth.se (M. Martinelle); mavem@kth.se (E. Malmström)

Table of Contents

Experimental **2**
- Lipase Catalyzed acylation of hydroxyl vinyl ether 2
- Synthesis of bifunctional vinyl ether terminated ester monomers for photopolymerization: 5
- Analytical Methods 6

Results **8**
- Synthesis of vinyl ether esters through enzyme Catalysis 8
- Control reactions 11
- Radical and Cationic Photopolymerrization of Vinyl Ether Ester Building Blocks 12

Experimental

Lipase Catalyzed acylation of hydroxyl vinyl ether

Reaction progression in different solvents and at different temperatures: The reaction coordinates were followed in bulk, toluene, acetonitrile (ACN), methyl tert butyl ether (MTBE) and 2-methyltetrahydrofuran (Me-THF). The carboxylic acid 10-undecenoic acid (UA) and the hydroxyl vinyl ether, either BVE or HVE, were added in a 1:1 ratio, see Table S1 for amounts of UA and BVE or HVE**.** For entry 1-5 in Table S1 molecular sieves, size 4Å, dodecane 60 µL, 0.26 mmol (used as internal standard) were mixed in 5 mL round bottom flasks with magnetic stirring. The reactions were run either in bulk (without solvent) or in 1 mL solvent (1.6 M for BVE and 1.3 M for HVE). The reactions were started by the addition of 10 wt% Novozyme 435. Consecutive samples were withdrawn, filtered through a cotton filter and analyzed with GC. Endpoint samples were further analyzed by ^{1}H-NMR.

Additionally, as described above, the reaction progression between UA and either BVE or HVE, (see Table S1 for amounts entry 1, 6-7), was studied in bulk at: 22 (room temperature), 60 and 90 °C, dodecane 73 µL, 0.3 mmol was used as internal standard.

Table S1. Reaction conditions for CalB catalyzed acyl transfer.

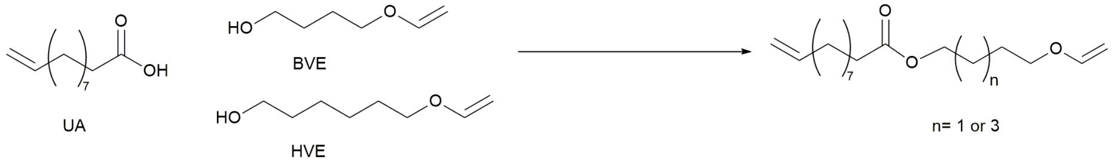

Entry	Acyl acceptor	Solvent	Temperature [°C]	Amount [b] [mmol]
1	BVE[a] / HVE[a]	-	22	1.7 / 2
2	BVE / HVE	Toluene	22	1.6 / 1.3
3	BVE / HVE	MTBE	22	1.6 / 1.3
4	BVE / HVE	Me-THF	22	1.6 / 1.3
5	BVE / HVE	ACN	22	1.6 / 1.3
6	BVE[a] / HVE[a]	-	60	2.3 / 2.6
7	BVE[a] / HVE[a]	-	90	2.3 / 2.6

[a] Catalyst-free reactions without CalB were run under these reaction conditions.

[b] Amount of acyl donor and acyl acceptor (1:1 molar ratio).

Reaction conditions for experiments with the guanidine base 1,5,7-Triazabicyclo[4.4.0]dec-5-ene (TBD) and the organometallic catalyst titanium(IV) butoxide ($Ti(OBu)_4$), Table S2.

Table S2. Reaction conditions for control reactions catalyzed by two conventional catalysts.

Entry	Catalyst	Amount of UA and BVE [mmol] [a]	Catalyst mol% [b]	Temperature [°C]	Time [min]
1	TBD	1.6	1.4	90	50
2	$Ti(OBu)_4$	3.2	0.5	160	56

[a] 1:1 molar ratio of acyl donor and acceptor

[b] mol% based on total monomer content

Acylation of hydroxyl vinyl ether with different carboxylic acids: The reactions were performed in gram scale. The acyl acceptor was mixed in a molar ratio 1:1 with the carboxylic acid (see Table S3 for amounts) with molecular sieves 4Å, in 5 mL round bottom flasks with magnetic stirring. The reactions were run either in bulk (without solvent) or in toluene-d_8 (1.1 M) at 22 ^oC. When lipoic acid was used in bulk the reaction was performed at 60 ^oC because lipoic acid did not dissolve in the vinyl ether monomer at ambient temperature. 11-Mercaptoundecenoic acid was not run in bulk because heating it above melting point caused a large increase in reaction rate between the thiol and the vinyl ether. The reactions were started by the addition of 10 wt% Novozyme 435 (of the sum of the monomers). The reactions were run for 1 h. Filtering off the enzyme using a cotton filter stopped the reactions. When the thiol-functional carboxylic acid 11-mercaptoundecanoic acid was used as acyl donor, approximately 2 wt% (total monomer weight) NPAL was added as a radical inhibitor. Reaction between UA and BVE or HVE were run in bulk, hence the product monomers were dissolved in acetone prior to filtration. The solvent (toluene and/or acetone) was removed from the filtrate under vacuum. Product monomers were analyzed by GC, ^{1}H-NMR, DEPT ^{13}C-NMR and FTIR.

Table S3. Reaction conditions used for the synthesis of vinyl ether esters.

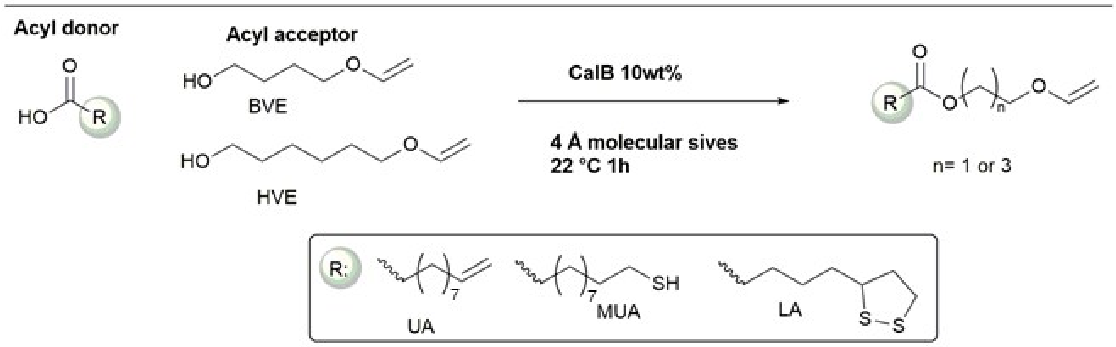

Vinyl ether functional ester	Solvent	Acyl donor / Acyl acceptor[a]	
		[mmol]	[M]
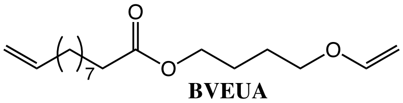 **BVEUA**	-	3.4	3.1
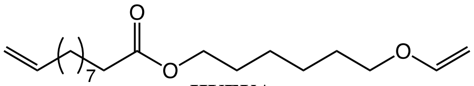 **HVEUA**	-	2.7	2.8
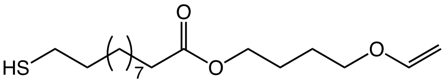 **BVEMUA**	Toluene	3.4	1.1
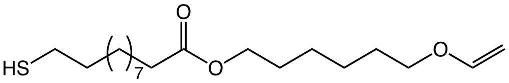 **HVEMUA**	Toluene	2.7	1.1
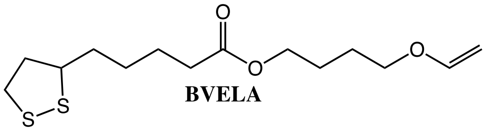 **BVELA**	Toluene	3.4	1.1
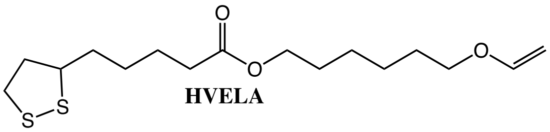 **HVELA**	Toluene	1.4	1.1

[a] molar ratio 1:1

Synthesis of bifunctional vinyl ether ester monomers for photo-polymerization:

Synthesis of HVEUA: The reactions were performed in gram scale. A slight excess of the acyl acceptor HVE was mixed with UA. The reactants were added to a 5 mL round bottom flasks with magnetic stirring and 4Å molecular sieves. The reaction was started by the addition of 10 wt% Novozyme 435 (of the sum of the monomers). After 45 min, in an open system with magnetic stirring, the temperature was raised to 95 °C and the pressure reduced to 50 mbar to evaporate the excess of HVE. The product was diluted in acetone and the enzyme was filtered of using a cotton filter stopped. Acetone was removed from the filtrate under vacuum.

Synthesis of HVEMUA: The reactions were performed on gram scale. The acyl acceptor HVE was mixed with MUA**,** exact stoichiometric equivalence of reagents was attempted. The reactants were mixed in toluene (1M of reactants) in a 10 mL round bottom flasks with magnetic stirring and 4Å molecular sieves. The reaction was started by the addition of 10 wt% Novozyme 435 (of the sum of the monomers). After 1 h filtering of the enzyme stopped the reaction. Toluene was removed from the filtrate under vacuum. Both HVEUA and HVEMUA were analyzed by GC, ^{1}H-NMR and FTIR.

Analytical Methods

^{1}H-NMR, ^{13}C-NMR and 2D (HMBC and HSQC) spectra were recorded on a Bruker AM 400 MHz instrument. The ^{1}H-NMR spectra were based on 16-40 scans. The ^{13}C-NMR spectra were based on 4096 scans; HMBC and HSQC were performed to confirm ^{1}H-NMR and ^{13}C-NMR and were based on 4 scans.

BVEUA:^{1}H-NMR ($CHCl_3$-d, δ reported in ppm): δ = 6.5 (dd, 1H, CH_2-C*H*O-), δ = 5.8 (m, 1H, CH_2C*H*CH_2-), δ = 4.9 (dd, 2H, CH_2CHCH_2-), δ = 4.2 (d, 1H, HC*H*CHO-), δ = 4.1 (t, 2H, -CH_2-OCO-), δ = 4.0 (d, 1H, *H*CHCHO-), δ = 3.7 (t, 2H, -CH_2-OCHCH_2), δ = 2.3 (t, 2H, -CH_2-CO-), δ = 2.0 (q, 2H, -CH_2CHCH_2), δ = 1.22-1.72 (m, aliphatic part). ^{13}C-NMR (CHCl3-d, δ reported in ppm): δ = 175 (-*C*OO-, from HMBC), δ = 152 (CH_2-*C*HO-), δ = 139 CH_2*C*H*C*H_2-), δ = 114

(CH_2CHCH_2-), δ = 86.6 (H*C*HCHO-), δ = 67.3 (-CH_2-OCHCH_2), δ = 63.9 (-CH_2-OCO-), δ = 34.4 (-CH_2CHCH_2), δ = 33.8 (-CH_2-COOCH_2-), δ = 19.8 - 29.1 (aliphatic part).

BVEMUA:^{1}H-NMR ($CHCl_3$-d, δ reported in ppm): δ = 6.5 (dd, 1H, CH_2-C*H*O-), δ = 4.2 (d, 1H, HC*H*CHO-), δ = 4.1 (t, 2H, -CH_2-OCO-), δ = 4.0 (d, 1H, *H*CHCHO-), δ = 3.7 (t, 2H, -CH_2-OCHCH_2), δ = 2.5 (q, 2H, SH-CH_2-), δ = 2.3 (t, 2H, -CH_2-COOCH_2-), δ = 1.8-1.2 (m, aliphatic part). ^{13}C-NMR ($CHCl_3$-d, δ reported in ppm): δ = 174 (-*C*OO-, from HMBC), δ = 152 (CH_2-*C*HO-), δ = 86.6 (H*C*HCHO-), δ = 67.3 (-CH_2-OCHCH_2), δ = 63.9 (-CH_2-OCO-), δ = 34 (-$CH_2$$CH_2$SH / -$CH_2$-CO-), δ = 29.4 - 19.6 (aliphatic part).

BVELA: ^{1}H-NMR ($CHCl_3$-d, δ reported in ppm): δ = 6.5 (dd, 1H, CH_2-C*H*O-), δ = 4.2 (d, 1H, HC*H*CHO-), δ = 4.1 (t, 2H, -CH_2-OCO-), δ = 4.0 (d, 1H, *H*CHCHO-), δ = 3.7 (t, 2H, -CH_2-OCHCH_2), δ = 3.6 (quint, 1H, >C*H*-CH_2), δ = 3.1 (m, 2H, -CH_2-SS-), δ = 2.5 (m, 1H, -HC*H*-CH_2SS-), δ = 2.3 (t, 2H, -CH_2-COOCH_2-), δ = 1.9 (m, 1H, -*H*CH-CH_2SS-), δ = 1.8-1.2 (m, aliphatic part). ^{13}C-NMR ($CHCl_3$-d, δ reported in ppm): δ = 174 (-*C*OO-, from HMBC), δ = 152 (CH_2-*C*HO-), δ = 86.6 (H*C*HCHO-), δ = 67.3 (-CH_2-OCHCH_2), δ = 63.9 (-CH_2-OCO-), δ = 56.3 (>*C*HCH_2-), δ = 40.2 (-CH-CH_2- CH_2-), δ = 38.5 (-CH_2-CH_2-SS-), δ = 29.4 - 19.6 (aliphatic part).

GC analysis was preformed on a Hewlett Packard 5890 SeriesII gas chromatofraph with a Agilent J&W CP-Sil 5 CB column (30 m x 0.25 mm). Inlet and detector temperature were set at 275 °C and 300 °C respectively. The temperature program stated at 80 °C for 1 min, increased by 5°/min to 160 °C kept for 1 min and then increased by 25 °C/min to 300 °C where it was kept for 3 min. The retention times were: BVE 4.4 min, HVE 8.2 min, dodecane 9.1 min, UA 15 min, MUA 21 min, BVEUA 23 min, HVEMUA 24 min.

FTIR spectra were recorded on a Perkin-Elmer Spectrum 2000 FT-IR instrument (Norwalk, CT) equipped with a single reflection (ATR: attenuated total reflection) accessory unit (Golden Gate) from Graseby Specac LTD (Kent, England) and a TGS detector using the Golden Gate setup. Data was collected at an optimized scanning rate of 1 scan 1.67 s^{-1} with a resolution of 4.0 cm^{-1}

Size exclusion chromatography (SEC) was performed on a Malvern VISCOTEK GPCmax equipped with a refractive index detector and TGuard column followed by two linear mixed bed columns (LT4000L) (35 °C). Tetrahydrofuran (THF) stabilized with BHT (1 mL/min) was used as mobile phase. The molecular weights were calculated against polystyrene standards (Polymer Laboratories, Mp = 1000 Da up to M = $4.5*10^6$ Da). All samples were filtered through a 0.2 µm PTFE filter (13 mm, PP housing, Alltech) before analysis.

UV-sources for curing of films was performed using a fusion UV curing system of model F300 equipped with fusion electrodeless bulbs standard type BF_9 which provided the dosage of 2.9 J/cm^2 measured in the wavelength interval 320-390 nm with a UVICURE Plus from Efsen Engineering. The light source used for the photo-RT-FTIR measurements was a Hamamatsu L5662 equipped with a standard medium-pressure 200 W L6722-01 Hg–Xe lamp and provided with optical fibers. The UV intensity was measured using a Hamamatsu UV-light power meter (model C6080-03) calibrated for the main emission line centered at 365 nm. The intensity was measured to be 30 mW cm^{-2}.

Differential scanning calorimetry (DSC) was performed with a Mettler Toledo differential scanning calorimeter DSC 820. Samples (mass 5-15 mg) were placed in 100 µL aluminum pans covered by aluminum lids. Mettler Toledo STARe software V9.2 was used to evaluate the results. Insert temperature was -60 °C and end point calibration was set to 5 min. Thereafter samples were heated from -60 °C to 150 °C at a rate of 10 °C/min with pure nitrogen (flow rate of 30 ml/min), equilibrated at 150 °C for 5 min, cooled to -60 °C and equilibrated for 5 min, thereafter heated again to 150°C

Results

Synthesis of vinyl ether esters through enzyme Catalysis

The rate constants (k_d) and the initial rate (in µmol converted monomer per µmol added catalyst and second) for reaction between **1a** and **2a** catalyzed by CalB in different solvents are shown in Table S5. k_d was calculated by assuming pseudo first order kinetic at the beginning of the reaction. k_d was calculated from equation 1:

$$\ln\left(\frac{[M]}{[M]_0}\right) = -kt$$

Where $[M]_0$ and $[M]$ are the concentrations of 10-Undecenoic acid at time zero and time t (in minutes).

Table S4. Rate constants of the acylation of BVE with UA in various solvents at 22 °C (room temperature).

Solvent	k_d [a] [min^{-1}]	Initial reaction rate [a] [$\mu mol_{monomer}*\mu mol_{CalB}*min^{-1}$]
Bulk	0.064	1500
Toluene	0.049	1200
MTBE	0.031	750
Me-THF	0.030	750
ACN	0.02	540

[a] calculated from 3.3 wt% active lipase on carriers [1]

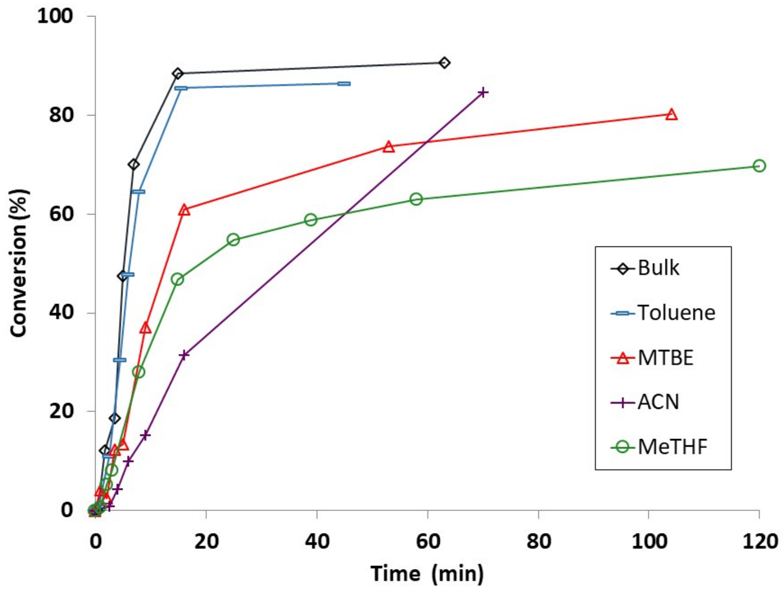

Figure S1. Reaction progress for acylation of BVE with UA in various solvents at 22°C (room temperature). Conversion of BVE was followed by GC.

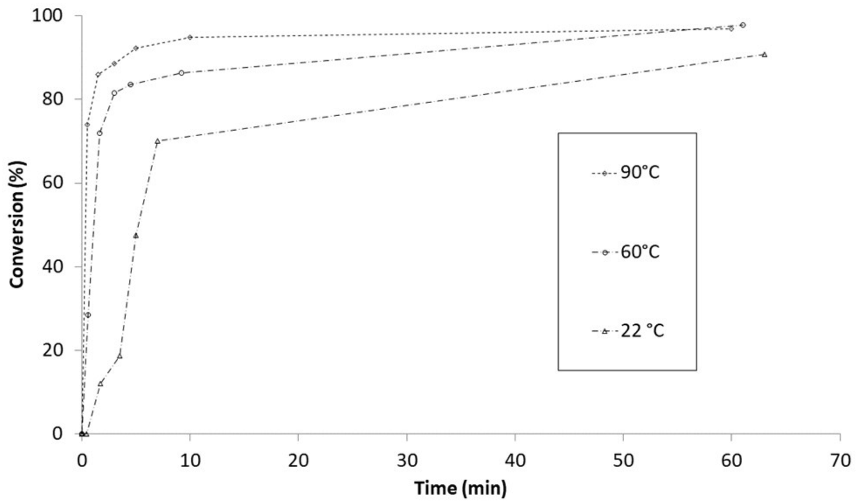

Figure S2. Reaction progress for acylation of BVE with UA at various temperatures. Conversion of BVE was followed by GC for the reaction at 22 and 60 °C and with ^{1}H-NMR for the reaction at 90 °C.

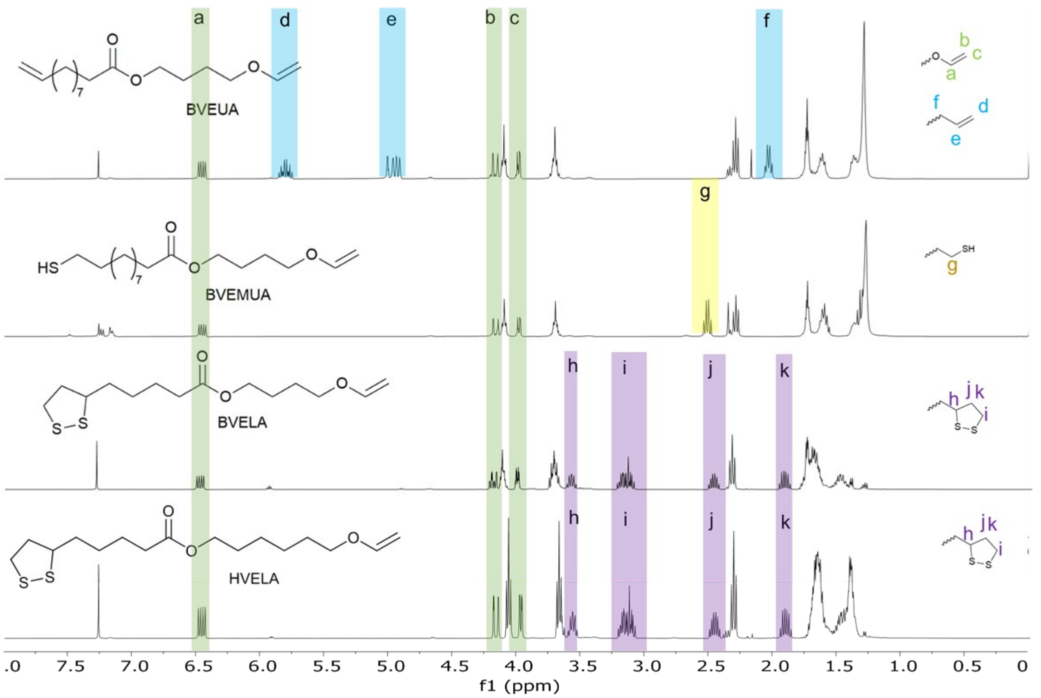

Figure S3. ^{1}H-NMR spectra of the synthesized monomers BVEUA, BVEMUA, BVELA and HVELA. HVEUA and HVEMUA can be seen in Figure 3, Figure S8 and S9 together with their respective polymers.

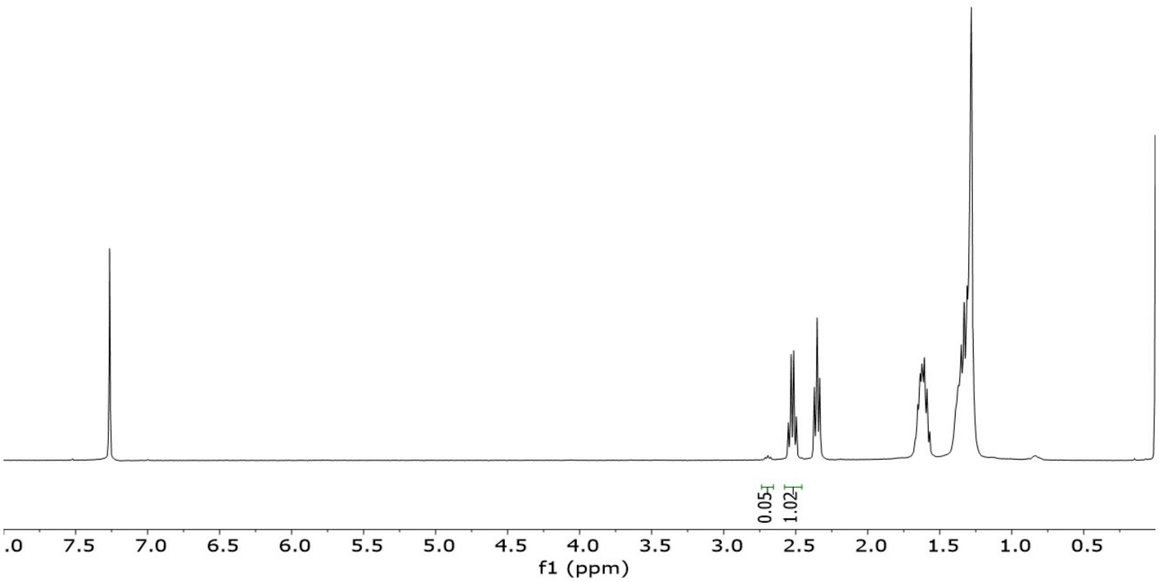

Figure S4. ^{1}H-NMR of MUA in $CHCl_3$-d. δ = 2.5 (q, 2H, SH-CH_2-), δ = 2.3 (t, 2H, -CH_2-$COOCH_2$-), δ = 1.8-1.2 (m, aliphatic part). δ = 2.7 shows that disulfides are

present in the monomer before ester synthesis is performed. The disulfide can also be seen in Figure S3 for BVEMUA, but the amount has not increased which means the formation is not caused by the ester reaction, but is already present from the beginning.

Control reactions

No acylation of hydroxyl vinyl ether with UA was observed without the addition of CalB. When TBD was used as a catalyst at 90 °C no ester formation was observed between UA and BVE. However, the vinyl ether functionality was rapidly consumed in the same way as the uncatalyzed reaction at 90 °C. Titanium-based catalysts are known to require high temperatures to be active, thus $Ti(OBu)_4$ was used as a catalyst at 160 °C. After 2.5 min at 160°C all vinyl ether functionality was gone (peaks at 6.5, 4.2 and 4.0 ppm, in 0 min spectrum). However, as can be seen in Figure S3 $Ti(OBu)_4$ still works as an esterification catalyst (formation of ester peak can be seen at 4.1 ppm), but the formed ester has no vinyl ether functionality and side products are formed.

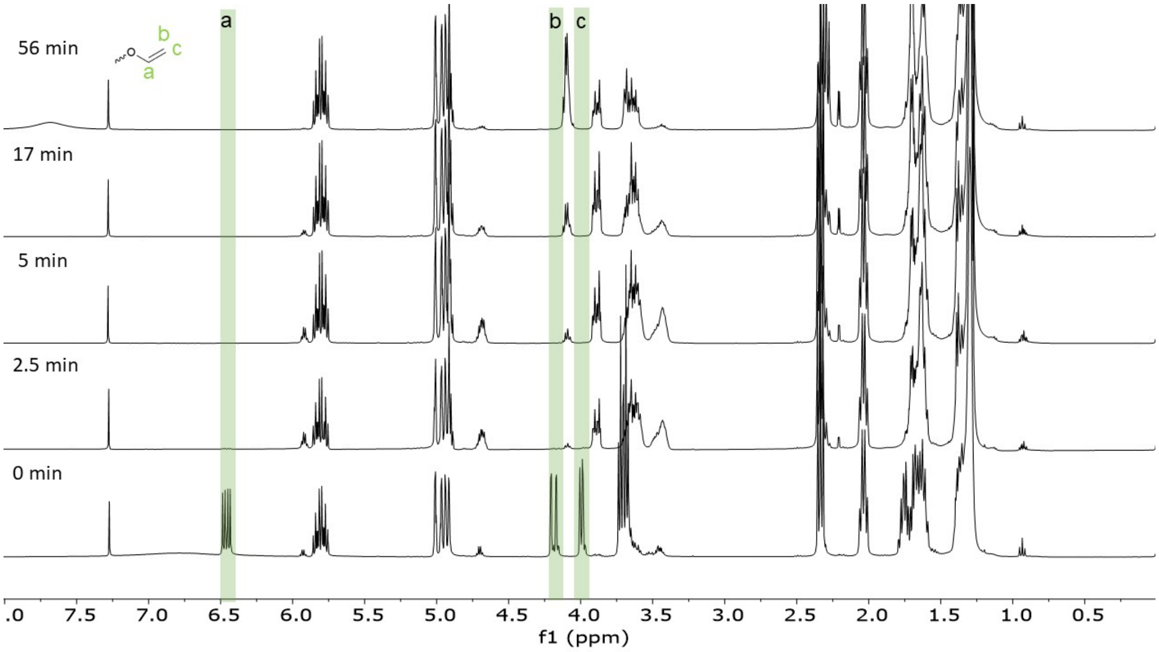

Figure S5 Controll reaction catalyzed by $Ti(OBu)_4$.

Radical and Cationic Photopolymerrization of Vinyl Ether Ester Building Blocks

GC spectra of the monomers HVEUA and HVEMUA that were used for photopolymerization are shown in Figure S6 and FigureS7.

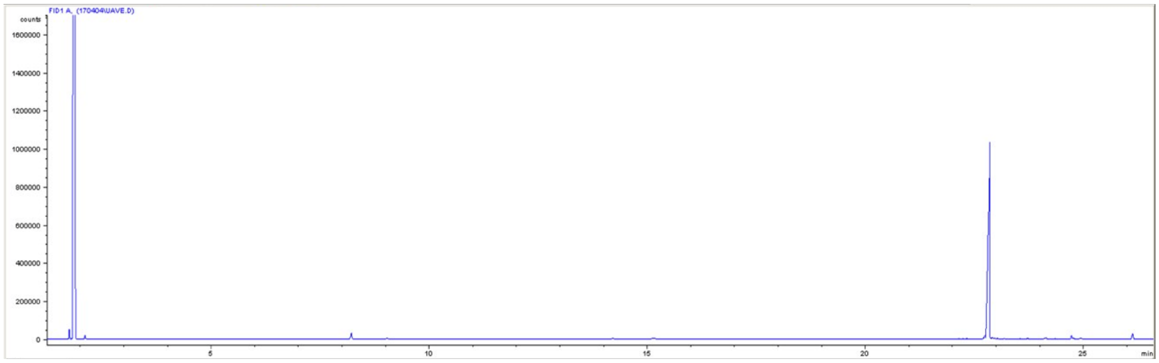

Figure S6. GC of HVEUA used for photopolymerization. The early peak is from the solvent, the peak at 9 min corresponds to unreacted monomer HVE and the peak around 23 min corresponds to the product monomer HVEUA.

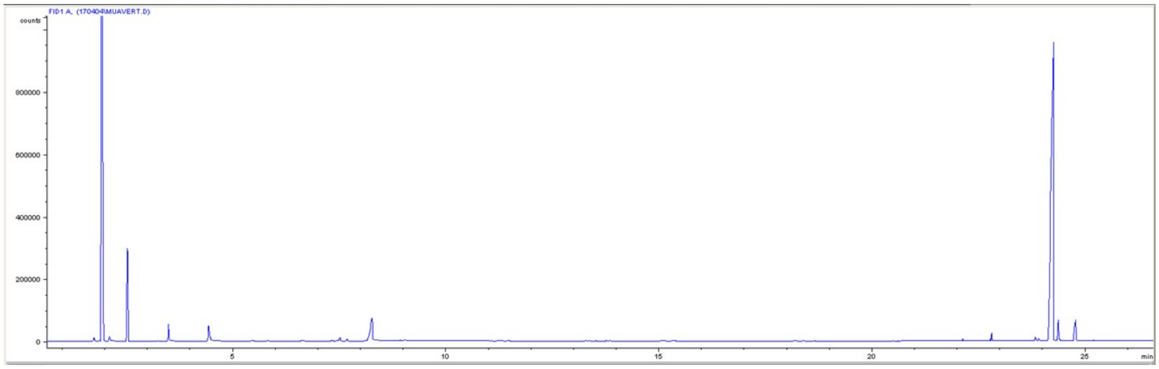

Figure S7 GC of HVEMUA used for photopolymerization. The early peak is from the solvent, the peak at 9 min corresponds to unreacted monomer HVE and the peak around 24 min corresponds to product monomer HVEUA.

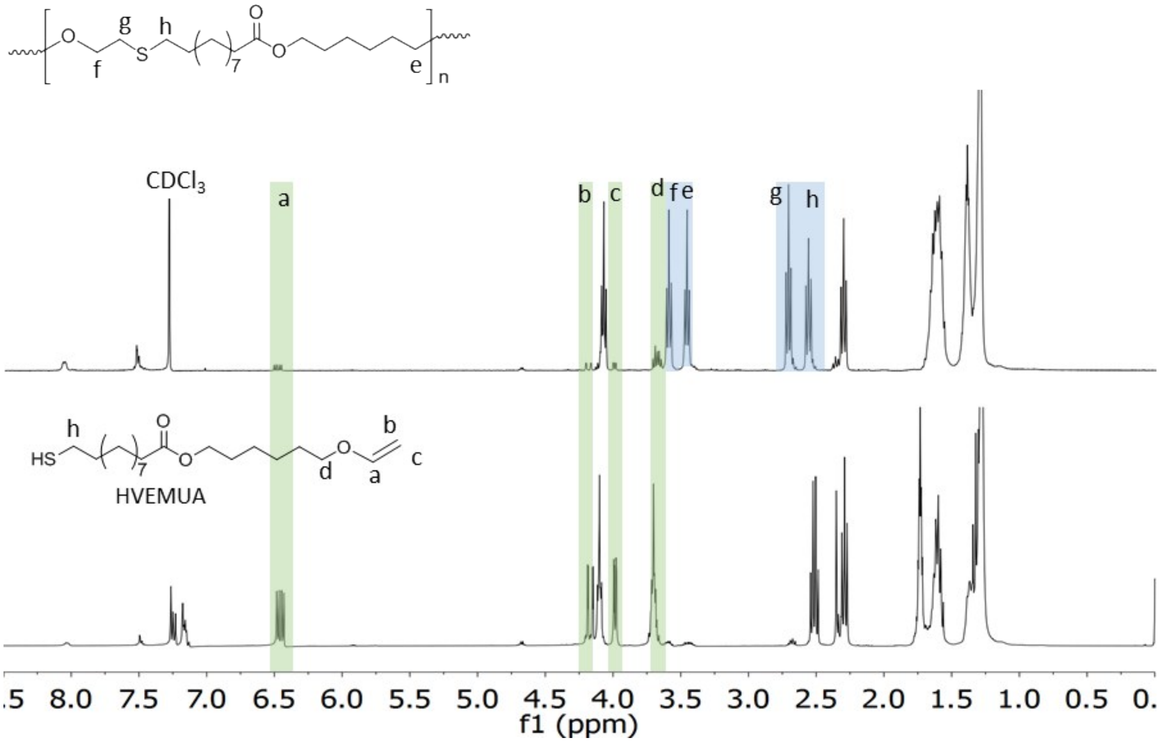

Figure S8. ^{1}H-NMR spectra before and after radical polymerization of HVEMUA. The lower spectrum is of the starting compound HVEMUA and the upper spectrum is from after radical polymerization of HVEMUA. The formation of the peaks denoted f, e, and g confirm the radical polymerization. No significant shift can be observed for h before and after polymerization.

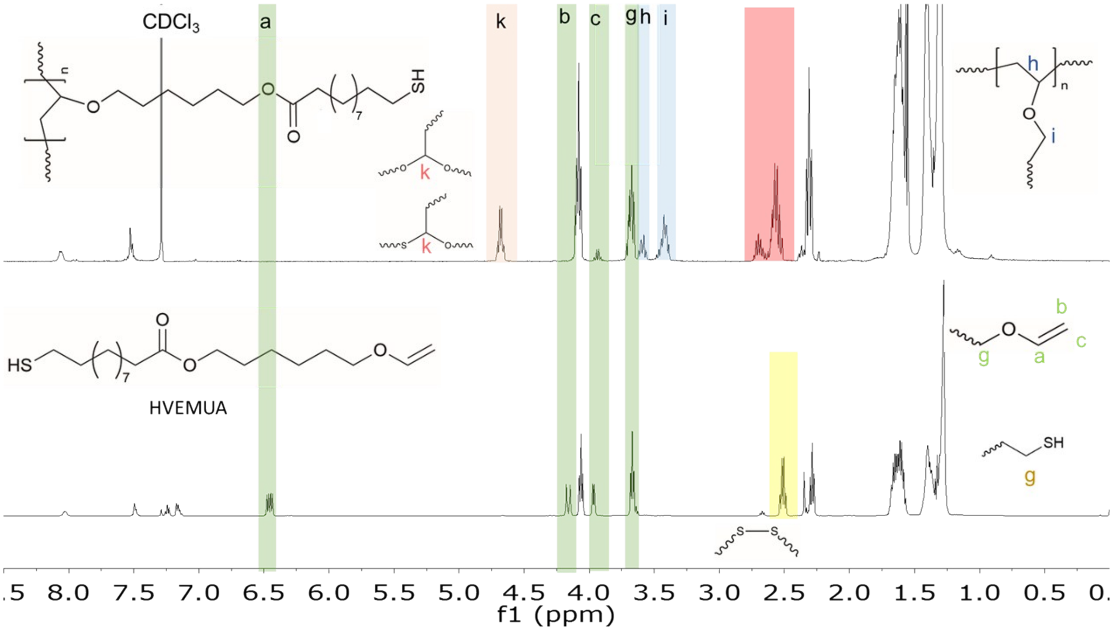

Figure S9. ^{1}H-NMR spectra before and after cationic polymerization of HVEMUA. The lower spectrum is of the starting compound HVEMUA and the upper spectrum is from after cationic polymerization of HVEMUA. The formation of the peaks denoted h and i show that some of the starting compound polymerizes cationically. However some radicalpolymerization of HVEMUA occures subsequentlly, which can be seen by the formation of the peaks in the region shaded in red (compare to Figure S8). In addition, the formation of a peak at 4.7 ppm (labeled k) is thought to be due to formation of thioacetal/polyacetal. At 8 and 7.5 ppm are from the radical scavenger, see Figure S11.

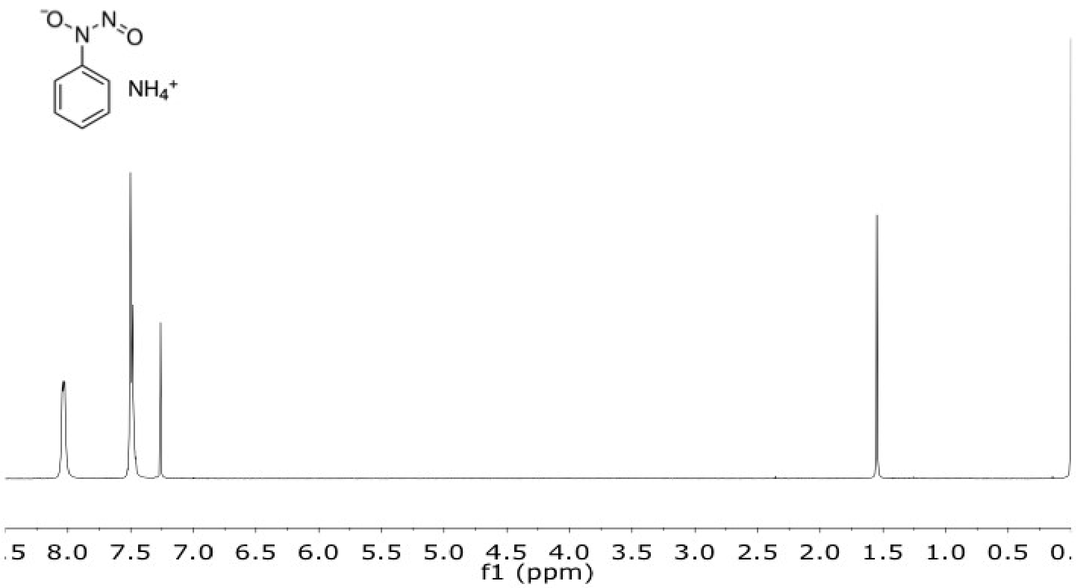

Figure S10. ^{1}H-NMR of the radical scavenger N-nitroso-N-phenylhydroxylamine aluminium complex. As can be seen the peaks at 7.5 ppm in Figure S8 and S9 correspond to shifts in from the radical scavenger and are not side products.

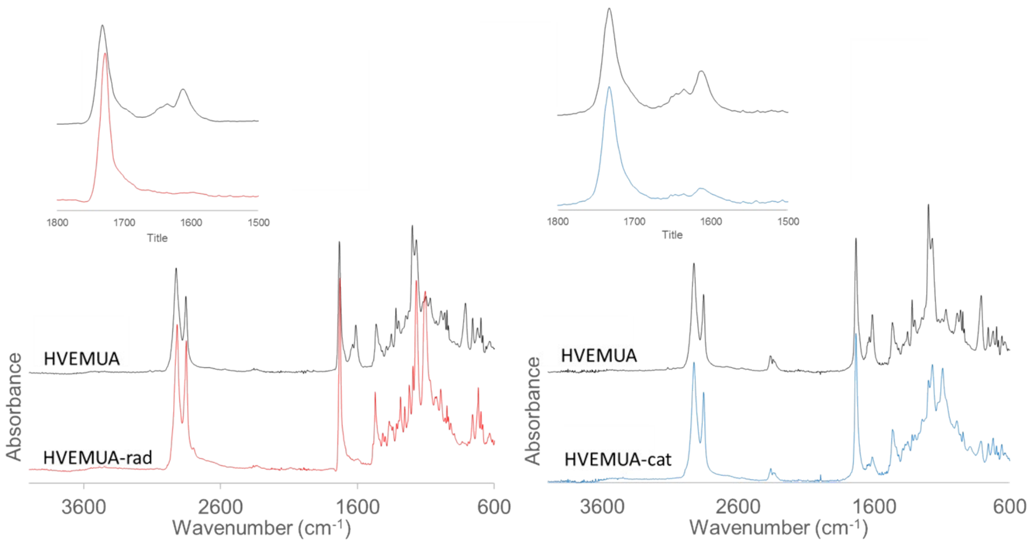

Figure S11. FTIR Spectra before (black spectra) and after (colored spectra) polymerization of HVEMUA. Left spectrum shows before and after radical polymerization and right spectrum shows before and after cationic polymerization. The zoomed region 1800 - 1600 cm^{-1} shows the carbonyl from the ester (1735 cm^{-1}) and the vinyl ether peaks (1613 cm^{-1}).
